# Drainage-Tube in the Chest for Twenty-One Months

**Published:** 1886-07-20

**Authors:** Oscar J. Coskery

**Affiliations:** Professor of Surgery, College of Physicians and Surgeons, Baltimore


### DRAINAGE-TUBE IN THE CHEST FOR TWENTY-ONE MONTHS.

BY OSCAR J. COSKERY, M. D.,

Professor of Surgery, College of Physicians and Surgeons, Baltimore. [Read before the Medical and Surgical Society of Baltimore.]

George C., aged 26, single, presented himself at St. Joseph's Hospital in July, 1885. He stated that he had a rubber tube in the right side of his chest which had been there since December, 1883, and that he wanted it removed; he was not prepared to come into hospital then, however, but would come later. He was of spare build, sallow, freckled-faced, with demi-blond hair and a light moustache; height, five feet eleven and a half inches; weight, one hundred and thirty-three pounds. He had weighed one hundred and fifty-nine pounds.

Admitted into hospital on September 21, 1885, with following history: In May, 1883, an incision was made in the right axillary line, between the eighth and ninth ribs, and a rubber drainage-tube introduced. This "fell out" on the next day, and a "silver" tube was put in. This also came away, and the wound closed rapidly. At the first insertion of the tube, on July 3, 1883, "many" ounces of pus, he says, escaped. After extrusion of silver tube nothing further was done in an operative way until December 7, 1883, when, under chloroform, a rubber tube was introduced at the site of the first operation, and one end brought out in the right nipple-line. Since then a constant discharge of pus has occurred at each end of the tube. One year ago he had anasarca for about four weeks. A month ago he noticed, for a few days, when he got up in the morning, that his face was "swollen."

Condition on admission: patient very anæmic; two openings found in right side of chest, through each of which a portion of the drainage tube was found projecting; granulations at each opening had rather firmly grasped the tube.

Physical signs: on right side the respiratory sounds over upper half in front and upper two-thirds at back, moderately free; over lower half in front and lower third at back, just recognizable; on left side of chest, respiration puerile everywhere, with prolonged expiratory murmur over upper half, back and front.

On percussion: right side from clavicle to one inch above nipple, clear; from there to nipple (in nipple-line), *dulled;* from nipple to lower border of costal cartilages, dulness complete; line of dulness, seven inches in right nipple-line; in right axillary line, seven inches; in right scapular line, seven and a half inches. No enlargement of liver could be made out.

On ocular inspection a great difference of the two sides of the chest could be noted; the right subclavicular fossa was much increased, while the left was bulged out; there was flattening of the whole right anterior chest-wall, and projection of the left; the intercostal spaces were very apparent, also, on the right side. Respirations thirty-two to the minute.

Measurements of chest in repose: right side, on nipple-line, fifteen and three-fourths inches; left side, on nipple-line, seventeen inches; at ensiform cartilage, right side, fifteen and one-fourth inches; at ensiform cartilage, left side, seventeen and one-fourth inches.

On September 27, 1885, after some little difficulty, but without enlarging the openings, the tube was withdrawn through the wound in the nipple-line; a very slight amount of granulation-hemorrhage followed.

Upon examining the urine next morning, it was found heavily loaded with albumen; pus has run freely from each of the openings where the drainage-tube was; he has never had a convulsion; sometimes has headache, and has several times had, as to-day (the day after the tube was removed), a "fainting-fit."

The patient was put upon iron and cod-liver oil. His appetite continued poor, and as diarrhœa made its appearance, the iron was stopped. The albumen in the urine, however, greatly decreased in quantity.

On October 12, 1885, he stated that the discharge had ceased from each opening (it had been to the estimated extent of about three-quarters of a pint daily since the removal of the tube), and that now his appetite had improved. Two days after this he complained that the wounds were again discharging freely, but that although he still felt a desire for food his mouth was so sore he could not take anything into it. A very extensive aphthous ulceration was found, greatest upon the right side, and involving both lingual and buccal mucous membranes. Diarrhœa, which had also improved for a time, returned, and the patient began to go down-hill. Without showing improvement at any time, the patient became dissatisfied, and was removed by his friends on October 21, 1885.

I learned that he died of exhaustive suppuration on November 23, 1885, the discharge continuing from both openings in about the same quantity to the end.

The points of interest in this case seem to be the length of time the tube was continuously worn, and the amount of albumen in the urine. It was at first thought that the latter might be due to organic changes in the kidney as a consequence of the continued suppuration; but the great decrease in the quantity of the albumen excreted under treatment, while the discharge of pus still continued, with a very slight interval, rather seemed to negative the idea.—*Philadelphia Medical Times.*

